# Ventilation and perceived exertion are sensitive to changes in exercise tolerance: arm+leg cycling vs. leg cycling

**DOI:** 10.3389/fphys.2023.1226421

**Published:** 2023-08-01

**Authors:** Andrea Nicolò, Michele Girardi, Ilenia Bazzucchi, Massimo Sacchetti, Francesco Felici

**Affiliations:** ^1^ Laboratory of Exercise Physiology, Department of Movement, Human and Health Sciences, University of Rome “Foro Italico”, Rome, Italy; ^2^ Division of Respiratory and Critical Care Physiology and Medicine, The Lundquist Institute for Biomedical Innovation at Harbor-UCLA Medical Center, Institute of Respiratory Medicine and Exercise Physiology, Torrance, CA, United States

**Keywords:** endurance performance, breathing control, respiratory frequency, incremental test, time to exhaustion, fatigue, breathing pattern, oxygen uptake

## Abstract

**Purpose:** Growing evidence suggests that respiratory frequency (*f*_R_) is a marker of physical effort and a variable sensitive to changes in exercise tolerance. The comparison between arm+leg cycling (Arm+leg) and leg cycling (Leg) has the potential to further test this notion because a greater exercise tolerance is expected in the Arm+leg modality. We systematically compared Arm+leg vs. Leg using different performance tests.

**Methods:** Twelve males underwent six performance tests in separate, randomized visits. Three tests were performed in each of the two exercise modalities, i.e. an incremental test and two time-to-exhaustion (TTE) tests performed at 90% or 75% of the peak power output reached in the Leg incremental test (PPO_Leg_). Exercise tolerance, perceived exertion, and cardiorespiratory variables were recorded during all the tests.

**Results:** A greater exercise tolerance (*p* < 0.001) was found for Arm+leg in the incremental test (337 ± 32 W vs. 292 ± 28 W), in the TTE test at 90% of PPO_Leg_ (638 ± 154 s vs. 307 ± 67 s), and in the TTE test at 75% of PPO_Leg_ (1,675 ± 525 s vs. 880 ± 363 s). Unlike $\dot{V}$ O_2_ and heart rate, both *f*_R_ and minute ventilation were lower (*p* < 0.003) at isotime in all the Arm+leg tests vs. Leg tests. Furthermore, a lower perceived exertion was observed in the Arm+leg tests, especially during the TTE tests (*p* < 0.001).

**Conclusion:** Minute ventilation, *f*_R_ and perceived exertion are sensitive to the improvements in exercise tolerance observed when comparing Arm+leg vs. Leg, unlike $\dot{V}$ O_2_ and heart rate.

## 1 Introduction

The comparison between different exercise modalities has the potential to improve our understanding of the physiology of endurance performance. Classical studies have compared leg cycling (Leg) with arm+leg cycling (Arm+leg) to unravel the mechanisms limiting maximal oxygen uptake (Astrand and Saltin, 1961; Secher et al., 1974; Bergh et al., 1976). Findings from these studies have contributed to outlining the important role of cardiocirculatory factors in setting the upper limit for maximal aerobic power. Indeed, the peak value of oxygen uptake ( $\dot{V}$ O_2_peak) is not always proportional to the differences in the amount of muscle mass involved in various exercise modalities, as the addition of arm work to leg work generally does not increase $\dot{V}$ O_2_peak more than about 5%–10% (Astrand and Saltin, 1961; Gleser et al., 1974; Secher et al., 1974; Secher and Volianitis, 2006). In fact, the comparison between arm+leg cycling and leg cycling is suitable for gaining insight into other physiological responses that have received less attention so far, including the variables associated with physical effort and changes in exercise tolerance. Exercise tolerance is here defined as the tolerated duration during a time-to-exhaustion (TTE) test performed at a constant work rate or the peak power output (PPO) achieved during an incremental test (Van De Walle and Vukovich, 2018). Some findings have shown that arm+leg cycling results in a greater exercise tolerance compared to leg cycling alone (arms hanging on the participant’s side) (Gleser et al., 1974; Secher et al., 1974; Nagle et al., 1984), but it is unclear if this difference is still evident when arm+leg cycling is compared to conventional leg cycling (Secher et al., 1974; Bergh et al., 1976; Nagle et al., 1984). When matched for the same absolute total power output, the greater exercise tolerance that might be expected for arm+leg cycling makes the comparison with (conventional) leg cycling valuable for testing the proposition that improvements in exercise tolerance are accompanied by consistent changes in the responses of respiratory frequency (*f*_R_) and perceived exertion (Nicolò and Sacchetti, 2023).

Growing evidence suggests that *f*_R_ is a valid marker of physical effort (Nicolò et al., 2014; Nicolò et al., 2016; Nicolò et al., 2017a; Nicolò et al., 2017b; Nicolò et al., 2019; Nicolò and Sacchetti, 2023) and that its time course reflects changes in exercise tolerance (Nicolò and Sacchetti, 2023). In a variety of conditions where exercise tolerance is reduced (experimentally) or lowered (in a cross-sectional comparison), the rate of increase in *f*_R_ is higher, both during incremental and TTE tests. On the other hand, the rate of increase in *f*_R_ is lower when assessing exercise strategies, experimental interventions or other conditions leading to an improvement in exercise tolerance (Nicolò and Sacchetti, 2023). The sensitivity of *f*_R_ to changes in exercise tolerance and the close association between *f*_R_ and perceived exertion are among the factors suggesting that *f*_R_ is to a large extent modulated by central command (the activity of motor and premotor brain areas relating to voluntary locomotor muscle contraction) during high-intensity exercise (Nicolò and Sacchetti, 2023). This explains why *f*_R_ can be considered a marker of physical effort, which is defined as the degree of motor effort (i.e. the magnitude of central command) (Nicolò et al., 2017b). However, measuring the magnitude of central command during “real” exercise conditions is particularly challenging. Hence, it is important to use different approaches (including the comparison of different exercise modalities) to provide indirect evidence on the contribution of central command to *f*_R_ modulation (Nicolò and Sacchetti, 2023).

The comparison between arm+leg cycling and leg cycling may either challenge or reinforce the notion that the increase in *f*_R_ during high-intensity exercise reflects changes in exercise tolerance and is influenced by central command. *f*_R_ is also modulated by muscle afferent feedback from groups III and IV (hereinafter muscle afferent feedback) (Dempsey et al., 2014; Girardi et al., 2021; Nicolò and Sacchetti, 2023), and this drive to breathe may have a greater relative contribution to *f*_R_ modulation when arm muscles assist leg muscles during arm+leg cycling, in view of a potentially larger amount of muscle mass concomitantly involved in exercise (Dempsey et al., 2014; Nicolò and Sacchetti, 2023). Indeed, even the passive movement of the legs leads to a substantial increase in *f*_R_ that is at least partially mediated by muscle afferent feedback (Girardi et al., 2021), and this drive to breathe may increase further when adding the movement of the upper limbs. The contribution of muscle afferent feedback to *f*_R_ may partially confound the association between *f*_R_ and perceived exertion because the latter is supposed to be largely independent of muscle afferent feedback (Marcora, 2009; Bergevin et al., 2023). While arm+leg cycling may also result in a higher $\dot{V}$ O_2_ compared to leg cycling at the same submaximal power output (Hoffman et al., 1996), *f*_R_ largely dissociates from metabolic rate and is not substantially modulated by metabolic inputs, unlike tidal volume (V_T_) (Nicolò et al., 2017a; 2018; Nicolò and Sacchetti, 2019; Nicolò et al., 2020a; Nicolò and Sacchetti, 2023). On the other hand, some findings seem to support the association between *f*_R_ and perceived exertion during both arm+leg cycling and leg cycling. Robertson et al. (1986) found similar responses of *f*_R_—but not V_T_—and the ratings of perceived exertion (RPE) during arm cycling, leg cycling and arm+leg cycling for intensities ranging from 20% to 80% of $\dot{V}$ O_2_peak. However, the authors neither reported the responses of *f*_R_ and RPE when exhaustion was approaching nor described if the two variables were sensitive to between-modality changes in exercise tolerance. Further studies are required to address this issue.

The purpose of the present study was to systematically assess whether exercise tolerance improves with arm+leg cycling vs. (conventional) leg cycling and whether *f*_R_ and perceived exertion are sensitive to the expected differences in exercise tolerance. To increase the robustness of our evaluation, we compared arm+leg cycling vs. leg cycling using two exercise paradigms (i.e. incremental test and TTE test) and three comparisons, as the TTE test was performed at two different intensities. We tested the hypotheses that i) arm+leg cycling improves exercise tolerance compared to leg cycling irrespective of the exercise paradigm; and ii) *f*_R_ is a good marker of physical effort sensitive to between-modality changes in exercise tolerance, unlike other physiological variables such as $\dot{V}$ O_2_ and heart rate (HR).

## 2 Materials and methods

### 2.1 Participants

Twelve recreationally trained males (mean ± SD: age 26 ± 4 years; stature 1.79 ± 0.08 m and body mass: 81 ± 10 kg) volunteered to participate in this study. The volunteers recruited participated in one or more sporting activities requiring the use of both arms and legs (e.g. rugby, extreme conditioning program training, and triathlon), as the benefits of arm+leg cycling vs. leg cycling may be more pronounced for individuals exercising with both upper and lower limbs (Secher et al., 1974). The study was approved by the Institutional Review Board of the University of Rome “Foro Italico” in compliance with the *Declaration of Helsinki* (CAR 07/2019). Written informed consent was obtained from all participants. They were asked to refrain from vigorous exercise and the consumption of alcohol and caffeine in the 24 h preceding each laboratory visit.

### 2.2 Experimental overview

Participants reported to the laboratory on 7 different occasions over a 4-week period, with visits separated by at least 48 h. On the first visit, participants were familiarised with the experimental procedures and tests. On the subsequent visits, participants performed three performance tests to exhaustion in each of the two exercise modalities, i.e. arm+leg cycling and leg cycling. The performance tests consisted of a step incremental test and two TTE tests performed at different intensities. Specifically, the incremental tests (Arm+leg_INC_ and Leg_INC_) were performed on visits 2 and 3, in random order. The PPO of the Leg_INC_ test (PPO_Leg_) was used to set the power output of the TTE tests (i.e. 90% and 75% of PPO_Leg_), which were performed on visits 4–7. The order of Arm+leg and Leg tests was always randomized, as well as the order of the TTE tests at 90% and 75% of PPO_Leg_. All the tests were performed on a multimodal ergometer custom-made by ORF s.r.l Magnetic Days^®^ (Arezzo, Italy) and specifically developed for performing this study. Exhaustion was defined as the decrease in pedaling cadence below 60 rpm, either with the legs or arms. All testing was completed in a laboratory with a room temperature of 23°C ± 1°C. A cooling fan was used during all the tests, and mechanical, physiological, and perceptual variables were recorded as detailed below.

### 2.3 Multimodal ergometer

**FIGURE 1:**
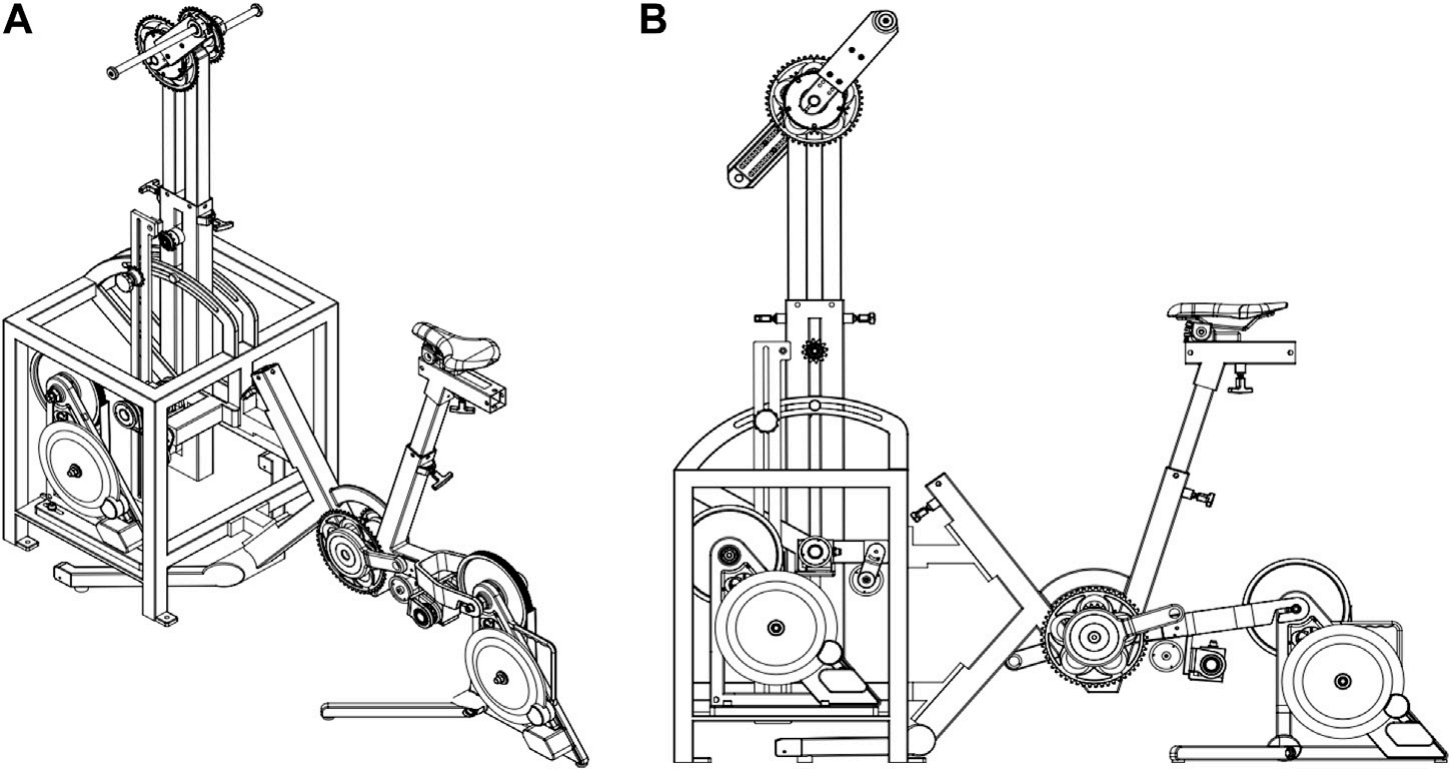
Three-dimensional **(A)** and lateral **(B)** views of the multimodal ergometer composed of a grinding ergometer and a cycling ergometer. The multimodal ergometer was custom-made by ORF s.r.l Magnetic Days^®^ (Arezzo, Italy). Note that each of the two ergometers is electromagnetically braked and is equipped with a professional torque transducer.

The multimodal ergometer was made up of a grinding ergometer and a cycling ergometer, and their combined use allowed the participants to perform arm+leg cycling (see Figure 1). Both the grinding and cycling ergometers were electromagnetically braked, equipped with professional torque transducers (model RT2A) certified by AEP transducers (Cognento, Modena, Italy), and calibrated according to the manufacturer’s instructions. The expanded uncertainty was lower than 0.114% for both torque transducers used. The arm cranks of the grinding ergometer were provided by Harken Italy SPA (Limido Comasco, Como). The chainring of the grinding ergometer was not mechanically connected with that of the cycling ergometer to allow for the separate measurement of the power output provided by the two ergometers. Hence, one of the advantages of this multimodal ergometer is the opportunity to set and register the contribution of arms and legs to the total power output. In all the Arm+leg tests, the relative contribution of the arms was initially set at 20% of the total power output based on the findings reported by Bergh et al. (1976). Thereafter, participants were allowed to request changes in the relative contribution of the arms throughout each test according to preference, and they were familiarised with this procedure on the first visit. The option of individualizing the relative contribution of the arms is supported by previous studies reporting inter-individual variability in preference (Hill et al., 2018) and exercise tolerance (Bergh et al., 1976) for different power output distributions between arms and legs. Participants were free to choose their preferred pedaling cadence in all the tests. In both Arm+leg and Leg modalities, the ergometer settings were set up on the first visit according to participants’ anthropometric characteristics and comfort, and were reproduced in the subsequent visits. The Arm+leg tests were performed on the multimodal ergometer, while the Leg tests were performed on the cycling ergometer (the participants were allowed to use the handlebars).

### 2.4 Step incremental tests

Before the incremental tests, a 5-min warm-up was performed to allow the participants to check the ergometer settings. For both Arm+leg_INC_ and Leg_INC_, the first stage of the incremental test consisted of 3 min at 150 W, and the power output was subsequently increased by 20 W every min. The power output of the first stage was chosen to ensure that at least 20% of the total power output could be sustained by the arms in the Arm+leg modality, and the minimum power output for the arm ergometer was about 25 W. Hence, it was not possible to select a power output lower than 150 W, and this limited the opportunity to rigorously determine the gas exchange threshold (GET) and the respiratory compensation point (RCP). The PPO reached in the incremental test was computed as the work rate of the last completed stage plus the fraction of time spent in the last uncompleted stage multiplied by the work-rate increment (i.e. 20 W). The Borg’s 6–20 scale (Borg, 1998) was used to collect ratings of perceived exertion (RPE) data every min. Participants were familiarised with the use of the RPE scale on the first visit and were asked to verbally provide an RPE value, as required by the Arm+leg modality. Breathing artifacts caused by speaking were then removed by data filtering, as described below. Participants did not receive any performance feedback or encouragement during any of the incremental tests performed.

### 2.5 Time to exhaustion tests

After a 10-min self-paced warm-up, participants performed a TTE test in each of the two exercise modalities (Arm+leg and Leg) and intensities (90% and 75% of PPO_Leg_) in separate visits. Hereinafter, the TTE tests are abbreviated as Arm+leg_TTE90_ (Arm+leg test at 90% of PPO_Leg_), Leg_TTE90_ (Leg test at 90% of PPO_Leg_), Arm+leg_TTE75_ (Arm+leg test at 75% of PPO_Leg_) and Leg_TTE75_ (Leg test at 75% of PPO_Leg_). Perceived exertion data were collected every min, while physiological and mechanical variables were measured continuously. Participants did not receive any performance feedback or encouragement during any of the TTE tests performed in this study.

### 2.6 Cardiorespiratory measures

*f*_R_, V_T_, minute ventilation ( $\dot{V}$ _E_), $\dot{V}$ O_2_, carbon dioxide output ( $\dot{V}$ CO_2_), end-tidal partial pressure of carbon dioxide (P_ETCO2_) and HR were measured breath-by-breath during all the tests using a metabolic cart (Quark CPET, Cosmed, Rome, Italy). The metabolic cart was calibrated following the manufacturer’s instructions.

### 2.7 Data analysis

Data were analyzed with MATLAB (R2016a, The Mathworks, Natick, MA, United States). The comparison of the physiological and perceptual responses between arm+leg cycling and leg cycling was performed in all the tests using the “individual isotime” analysis described by Nicolò et al. (2019). This analysis allows for between-condition comparisons while avoiding the data loss that occurs when the variability in TTE is not addressed on an individual basis (Nicolò et al., 2019). Briefly, breath-by-breath data of *f*_R_, V_T_, $\dot{V}$ _E_, $\dot{V}$ O_2_, $\dot{V}$ CO_2_, P_ETCO2_ and HR were filtered for errant breaths by deleting values greater than 3 standard deviations from the local mean (Lamarra et al., 1987). Subsequently, breath-by-breath data were linearly interpolated and extrapolated every second. Data were then smoothed by a moving average of 60 s. Likewise, RPE data collected every min were linearly interpolated and extrapolated every second. Thereafter, for each individual, the shortest test of each Arm+leg vs. Leg comparison (Arm+leg_INC_ vs. Leg_INC_, Arm+leg_TTE90_ vs. Leg_TTE90_ and Arm+leg_TTE75_ vs. Leg_TTE75_ were compared separately) was segmented into ten timepoints, and the same segmentation was used for the longest test of the same participant. This procedure was performed for all the participants as further detailed by Nicolò et al. (2019).

When reporting the relationship between different variables (i.e. RPE vs. *f*_R_, RPE vs. $\dot{V}$ _E_, RPE vs. HR, $\dot{V}$ _E_ vs. V_T_, $\dot{V}$ _E_ vs. $\dot{V}$ CO_2_, V_T_ vs. $\dot{V}$ CO_2_, and *f*_R_ vs. $\dot{V}$ CO_2_), another analysis called “relative isotime” was used as previously suggested (Nicolò et al., 2019). This analysis segments each test into ten timepoints based on the TTE of the test analyzed, and thus results in no data loss for any of the tests.

### 2.8 Statistical analysis

Statistical analyses were conducted using IBM SPSS Statistics 20 (SPSS Inc., Chicago, IL, United States). Data were checked for normality prior to analysis. A paired Student’s *t*-test was used to compare the performance values of Arm+leg_INC_ vs. Leg_INC_, Arm+leg_TTE90_ vs. Leg_TTE90_ and Arm+leg_TTE75_ vs. Leg_TTE75_ separately. The Cohen’s d effect size for paired *t*-test was then calculated and considered small, moderate or large for values ≥0.2, ≥0.5 and ≥0.8 respectively. A paired Student’s *t*-test was also used to compare the end-test values of physiological variables between Arm+leg and Leg tests. A two-way repeated-measures ANOVA (condition × time) was used to compare physiological and perceptual responses (processed with the “individual isotime” method) of Arm+leg vs. Leg for each of the three performance tests separately. When the sphericity assumption was violated, the Greenhouse–Geisser adjustment was performed. Partial eta squared (*η*_p_^2^) effect sizes were calculated for the main effect of condition, the main effect of time, and the interaction; *η*_p_^2^ values ≥0.01, ≥0.059 and ≥0.138 indicate small, medium and large effects respectively (Cohen, 1988). When a significant interaction was found, pairwise comparisons were performed at each time point using a one-way repeated measures ANOVA to identify differences between Arm+leg and Leg tests. The HR data of the incremental tests and the TTE tests at 75% of PPO_Leg_ were not normally distributed and were analyzed using Friedman’s two-way ANOVA. When statistical significance was found, this test was followed up by a Wilcoxon Signed-Rank test to identify where differences between Arm+leg and Leg tests occurred.

After processing data with the “relative isotime” method, the correlations between RPE and *f*_R_, RPE and $\dot{V}$ _E_, and RPE and HR were analyzed using a previously described method that adjusts for repeated observations within participants (Bland and Altman, 1995). A correlation coefficient (*r*) and a *p*-value were obtained by considering all the performance tests together. A *p*-value < 0.05 was considered statistically significant in all analyses. The results are expressed as mean ± SD in the text and as mean ± SE in the Figures.

## 3 Results

### 3.1 Step incremental tests

**FIGURE 2:**
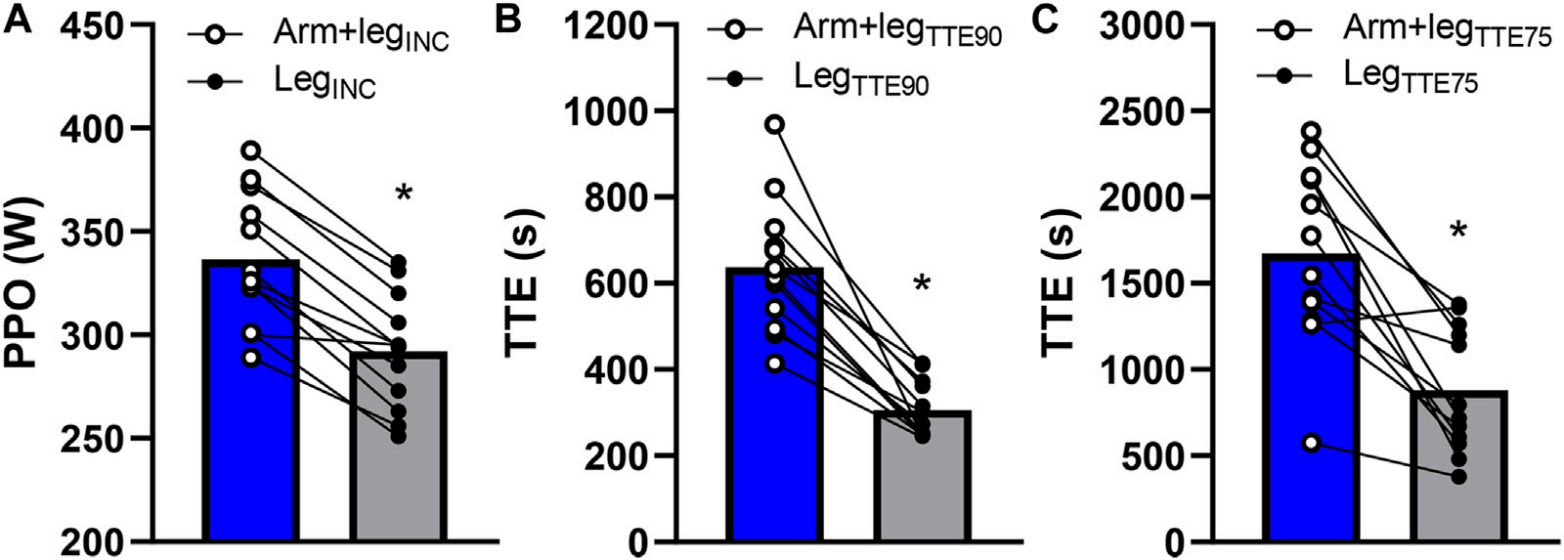
Average performance differences between Arm+leg tests (blue bar graphs) and Leg tests (grey bar graphs) for the incremental test **(A)**, the TTE test at 90% of PPO_Leg_ **(B)**, and the TTE test at 75% of PPO_Leg_ **(C)**. The open circles and the filled circles represent individual data during Arm+leg and Leg tests respectively. *p < 0.05 vs. Arm+leg.

**FIGURE 3:**
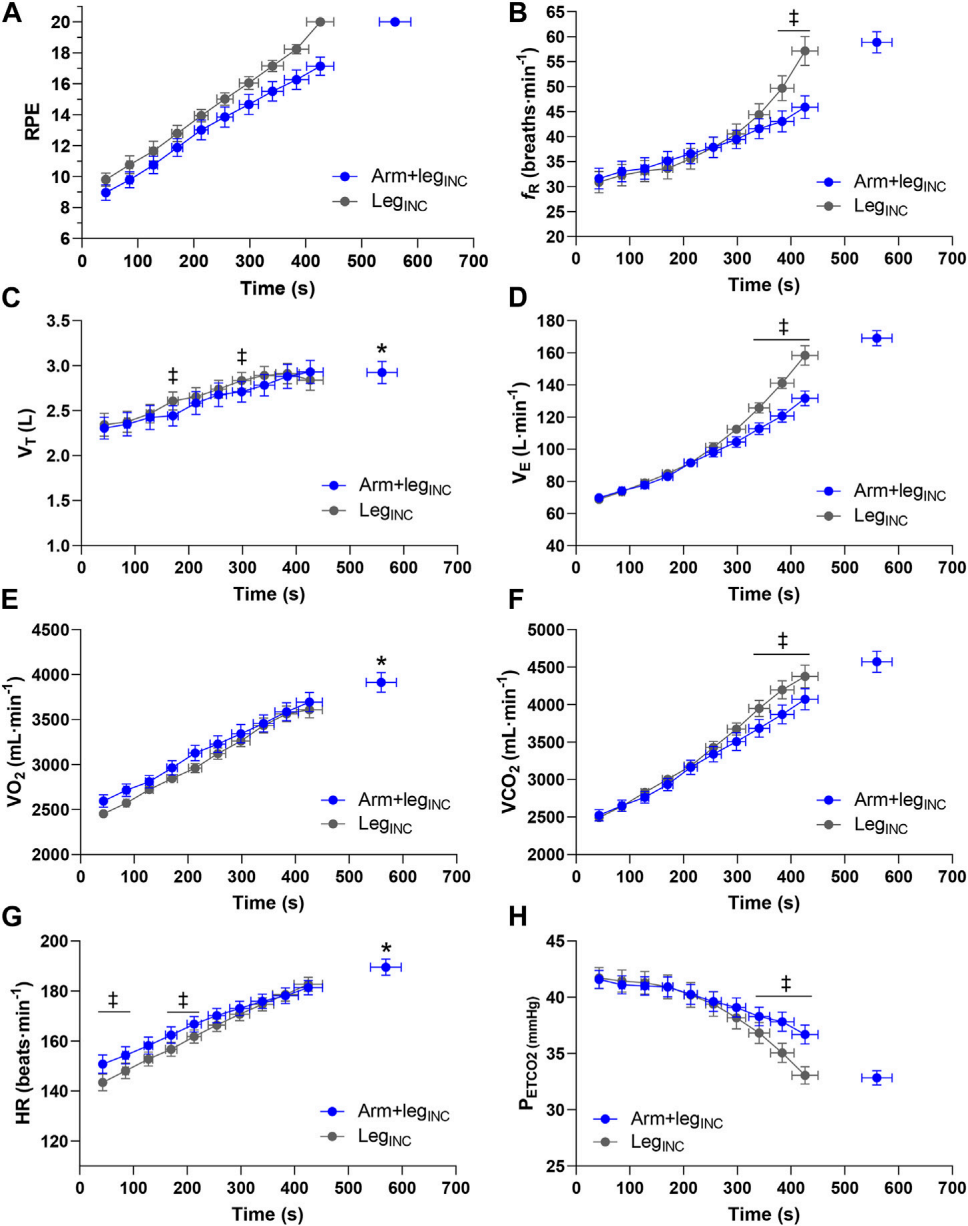
Group mean response of ratings of perceived exertion **(A)**, respiratory frequency **(B)**, tidal volume **(C)**, minute ventilation **(D)**, oxygen uptake **(E)**, carbon dioxide output **(F)**, heart rate **(G)** and end-tidal partial pressure of carbon dioxide **(H)** for Arm+leg_INC_ (blue circles) and Leg_INC_ (grey circles). ‡*p* < 0.05 vs. Arm+leg_INC_, *p < 0.05 vs. Leg_INC_.

A significantly greater (*p* < 0.001; Cohen’s d = 2.78) PPO was found in Arm+leg_INC_ (337 ± 32 W) vs. Leg_INC_ (292 ± 28 W) (Figure 2A). The average relative contribution of the arms to the total power output was 22% ± 2% in the Arm+leg_INC_ test. A higher pedaling cadence (*p* < 0.01) was found in the Leg_INC_ (84 ± 6 rpm) compared to that of the arms (76 ± 6 rpm) and legs (77 ± 7 rpm) of the Arm+leg_INC_ test. The two tests (Arm+leg_INC_ vs. Leg_INC_) showed significant differences (*p* < 0.043) in the end-test values of $\dot{V}$ O_2_ (3,913 ± 378 vs. 3,610 ± 310 mL min^−1^), HR (186 ± 11 vs. 181 ± 10 beats min^−1^), and V_T_ (2.92 ± 0.42 vs. 2.84 ± 0.40 L). When comparing the time course of the physiological and perceptual responses between Arm+leg_INC_ and Leg_INC_, a significant (*p* < 0.001) condition × time interaction was observed for *f*_R_ (*η*_p_^2^ = 0.75), $\dot{V}$ _E_ (*η*_p_^2^ = 0.74), $\dot{V}$ CO_2_ (*η*_p_^2^ = 0.48), V_T_ (*η*_p_^2^ = 0.22) and P_ETCO2_ (*η*_p_^2^ = 0.71). Statistically significant differences (*p* < 0.001) between Arm+leg_INC_ and Leg_INC_ were also found when evaluating the time course of HR. Figure 3 shows where a simple main effect of condition was found. All the variables reported in Figure 3 showed a main effect of time (*p* < 0.001; *η*_p_^2^ > 0.71). No main effect of condition was found for any of the variables, but some showed *p* < 0.1, i.e. RPE (*p* = 0.057), $\dot{V}$ _E_ (*p* = 0.055), $\dot{V}$ O_2_ (*p* = 0.091), and P_ETCO2_ (*p* = 0.091). Due to technical problems, HR analysis was performed for 11 participants.

### 3.2 TTE tests at 90% of PPO_Leg_

**FIGURE 4:**
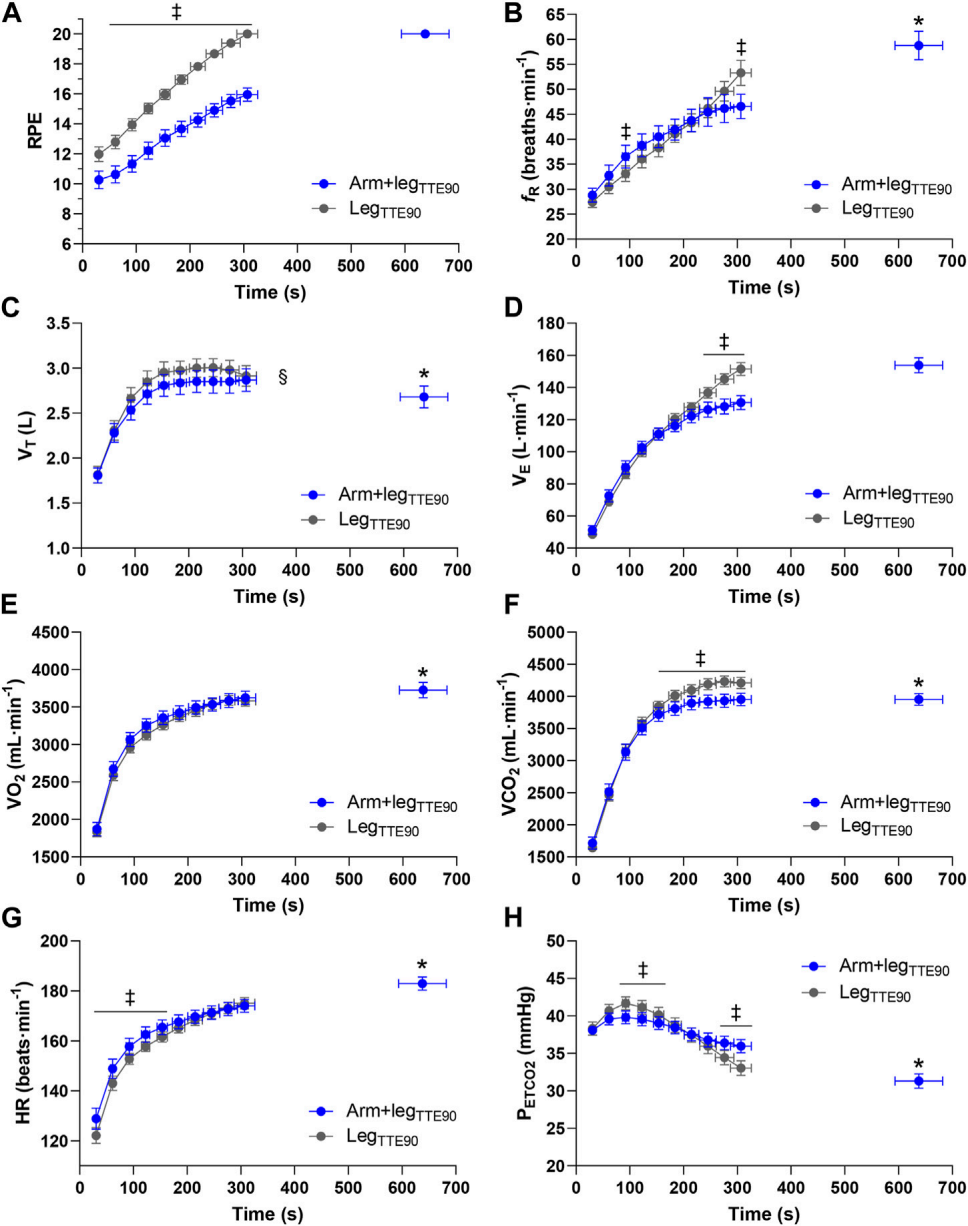
Group mean response of ratings of perceived exertion **(A)**, respiratory frequency **(B)**, tidal volume **(C)**, minute ventilation **(D)**, oxygen uptake **(E)**, carbon dioxide output **(F)**, heart rate **(G)** and end-tidal partial pressure of carbon dioxide **(H)** for Arm+leg_TTE90_ (blue circles) and Leg_TTE90_ (grey circles). ‡*p* < 0.05 vs. Arm+leg_TTE90_, *p < 0.05 vs. Leg_TTE90_, § main effect of condition.

A significantly longer (*p* < 0.001; Cohen’s d = 2.27) TTE was found in Arm+leg_TTE90_ (638 ± 154 s) vs. Leg_TTE90_ (307 ± 67 s) (Figure 2B). The average relative contribution of the arms to the total power output was 22% ± 2% in the Arm+leg_TTE90_ test. A higher pedaling cadence (*p* < 0.033) was found in the Leg_INC_ (83 ± 7 rpm) compared to that of the arms (73 ± 13 rpm) and legs (76 ± 6 rpm) of the Arm+leg_INC_ test. The two tests (Arm+leg_TTE90_ vs. Leg_TTE90_) showed significant differences (*p* < 0.034) in the end-test values of *f*_R_ (59 ± 10 vs. 53 ± 9 breaths min^−1^), $\dot{V}$ O_2_ (3,727 ± 361 vs. 3,581 ± 236 mL min^−1^), $\dot{V}$ CO_2_ (3,953 ± 319 vs. 4,209 ± 302 mL min^−1^), HR (183 ± 9 vs. 175 ± 8 beats min^−1^), V_T_ (2.68 ± 0.42 vs. 2.91 ± 0.40 L) and P_ETCO2_ (31 ± 3 vs. 33 ± 3 mmHg). When comparing the time course of the physiological and perceptual responses between Arm+leg_TTE90_ and Leg_TTE90_, a significant (*p* < 0.017) condition × time interaction was observed for RPE (*η*_p_^2^ = 0.32), *f*_R_ (*η*_p_^2^ = 0.52), $\dot{V}$ _E_ (*η*_p_^2^ = 0.67), $\dot{V}$ CO_2_ (*η*_p_^2^ = 0.60), HR (*η*_p_^2^ = 0.59) and P_ETCO2_ (*η*_p_^2^ = 0.66). $\dot{V}$ O_2_ showed *p* = 0.096. Figure 4 shows where a simple main effect of condition was found. All the variables reported in Figure 4 showed a main effect of time (*p* < 0.001; *η*_p_^2^ > 0.76), while a main effect of condition (*p* < 0.037) was found for RPE (*η*_p_^2^ > 0.72), $\dot{V}$ CO_2_ (*η*_p_^2^ > 0.40) and V_T_ (*η*_p_^2^ > 0.34); *p* = 0.088 was found for HR.

### 3.3 TTE tests at 75% of PPO_Leg_

**FIGURE 5:**
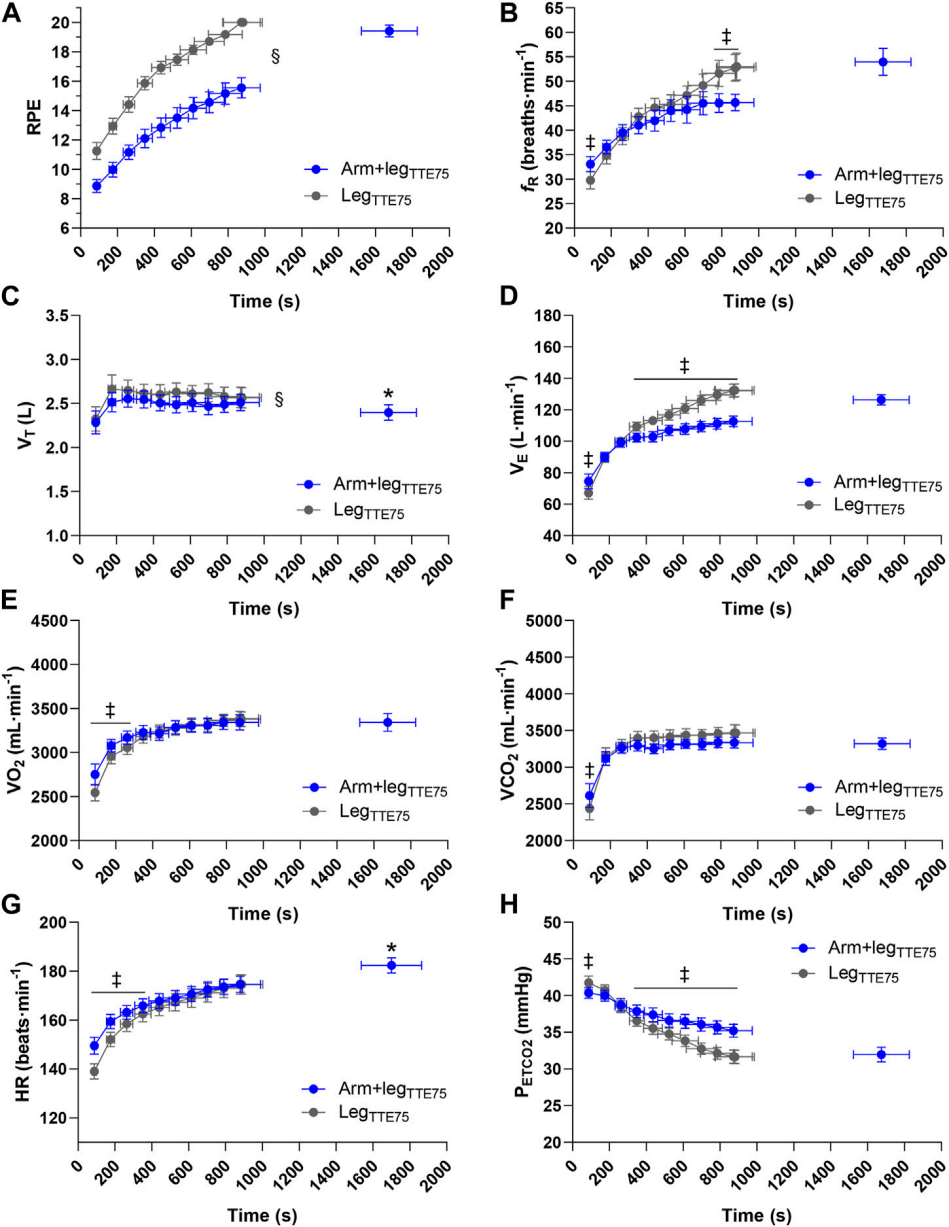
Group mean response of ratings of perceived exertion **(A)**, respiratory frequency **(B)**, tidal volume **(C)**, minute ventilation **(D)**, oxygen uptake **(E)**, carbon dioxide output **(F)**, heart rate **(G)** and end-tidal partial pressure of carbon dioxide **(H)** for Arm+leg_TTE75_ (blue circles) and Leg_TTE75_ (grey circles). ‡*p* < 0.05 vs. Arm+leg_TTE75_, *p < 0.05 vs. Leg_TTE75_, § main effect of condition.

A significantly longer (*p* < 0.001; Cohen’s d = 1.53) TTE was found in Arm+leg_TTE75_ (1,675 ± 525 s) vs. Leg_TTE75_ (880 ± 363 s) (Figure 2C). The average relative contribution of the arms to the total power output was 21% ± 1% in the Arm+leg_TTE75_ test. A higher pedaling cadence (*p* < 0.016) was found in the Leg_INC_ (80 ± 7 rpm) compared to that of the arms (73 ± 8 rpm) and legs (75 ± 6 rpm) of the Arm+leg_INC_ test. The two tests (Arm+leg_TTE75_ vs. Leg_TTE75_) also showed significant differences (*p* < 0.042) in the end-test values of HR (182 ± 10 vs. 175 ± 13 beats min^−1^) and V_T_ (2.40 ± 0.30 vs. 2.56 ± 0.40 L), while *p* = 0.084 was found for the end-test values of $\dot{V}$ _E_ (126 ± 11 vs. 132 ± 14 L min^−1^) and $\dot{V}$ CO_2_ (3,321 ± 270 vs. 3,465 ± 384 mL min^−1^); no significant differences (*p* = 0.57) were found for the end-test values of $\dot{V}$ O_2_ (3,342 ± 348 and 3,379 ± 294 mL min^−1^). When comparing the time course of the physiological and perceptual responses between Arm+leg_TTE75_ and Leg_TTE75_, a significant (*p* < 0.003) condition × time interaction was observed for *f*_R_ (*η*_p_^2^ = 0.41), $\dot{V}$ _E_ (*η*_p_^2^ = 0.67), $\dot{V}$ O_2_ (*η*_p_^2^ = 0.35), $\dot{V}$ CO_2_ (*η*_p_^2^ = 0.39), and P_ETCO2_ (*η*_p_^2^ = 0.65). RPE showed *p* = 0.068. Statistically significant differences (*p* < 0.001) between Arm+leg_TTE75_ and Leg_TTE75_ were also found when evaluating the time course of HR. Figure 5 shows where a simple main effect of condition was found. All the variables reported in Figure 5 showed a main effect of time (*p* < 0.021; *η*_p_^2^ > 0.31), while a main effect of condition (*p* < 0.011) was found for RPE (*η*_p_^2^ = 0.77), $\dot{V}$ _E_ (*η*_p_^2^ = 0.48), V_T_ (*η*_p_^2^ = 0.53) and P_ETCO2_ (*η*_p_^2^ = 0.47). Due to technical problems, HR analysis was performed for 11 participants.

### 3.4 The performance tests considered together

**FIGURE 6:**
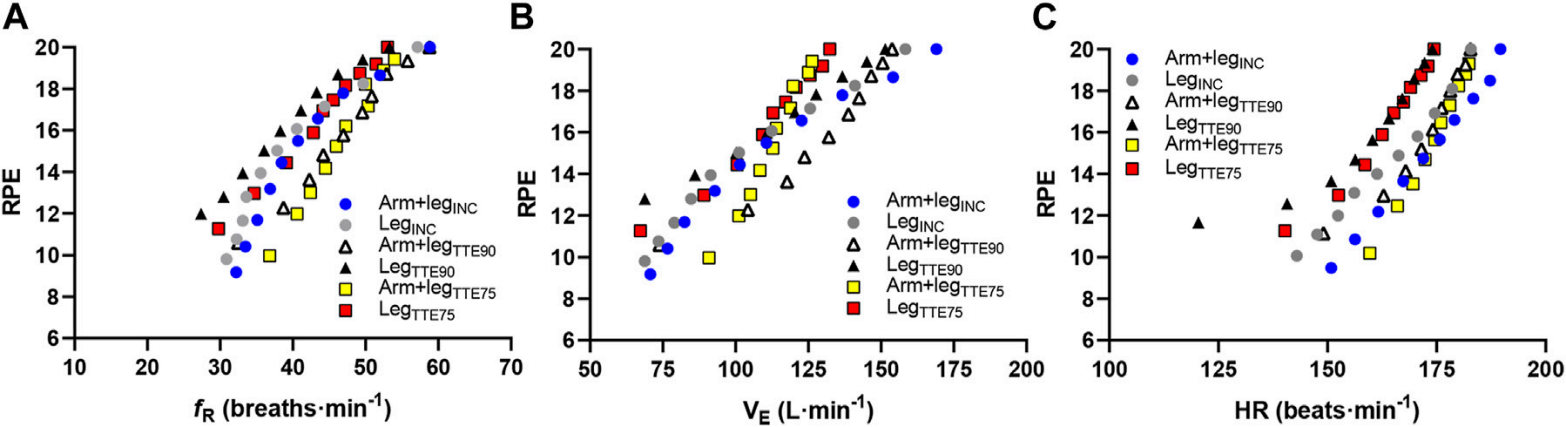
Correlations between RPE and respiratory frequency **(A)**, RPE and minute ventilation **(B)**, and RPE and heart rate **(C)** for the Arm+leg_INC_ (blue circles), Leg_INC_ (grey circles), Arm+leg_TTE90_ (open triangles), Leg_TTE90_ (black triangles), Arm+leg_TTE75_ (yellow squares) and Leg_TTE75_ (red squares). Each symbol represents the mean value of all participants at each percentage of the TTE.

When the performance tests were considered together, a significant correlation was found between *f*_R_ and RPE (*p* < 0.001; *r* = 0.75), HR and RPE (*p* < 0.001; *r* = 0.69), and $\dot{V}$ _E_ and RPE (*p* < 0.001; *r* = 0.80). A graphical representation of the correlations between these variables is depicted in Figure 6.

**FIGURE 7:**
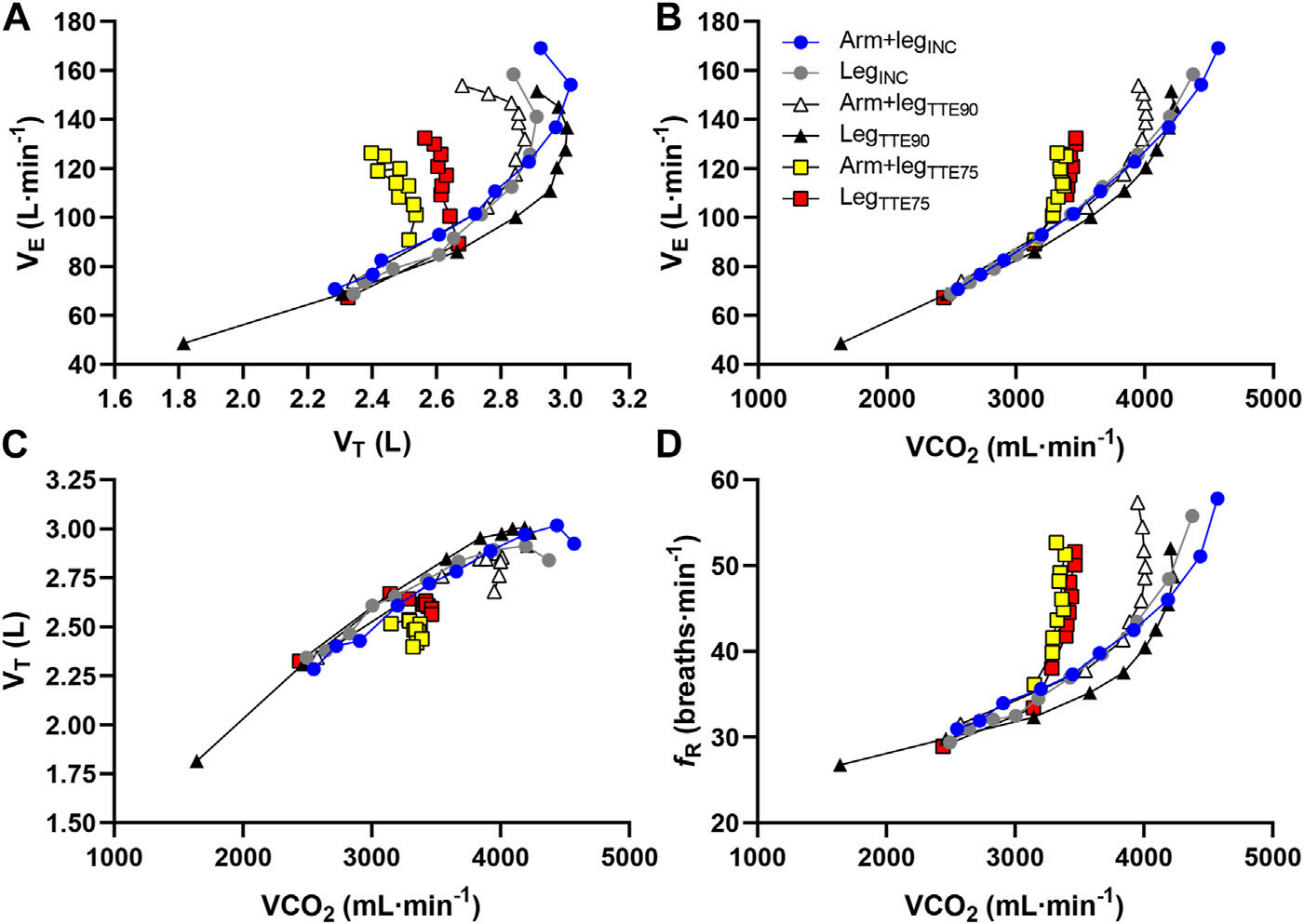
Relationship between minute ventilation and tidal volume **(A)**, minute ventilation and carbon dioxide output **(B)**, tidal volume and carbon dioxide output **(C)**, and respiratory frequency and carbon dioxide output **(D)** for the Arm+leg_INC_ (blue circles), Leg_INC_ (grey circles), Arm+leg_TTE90_ (open triangles), Leg_TTE90_ (black triangles), Arm+leg_TTE75_ (yellow squares) and Leg_TTE75_ (red squares). Each symbol represents the mean value of all participants at each percentage of the TTE.

Figure 7 shows the average response of the group when expressing $\dot{V}$ _E_ as a function of V_T_ values, and $\dot{V}$ _E_, V_T_ and *f*_R_ as a function of $\dot{V}$ CO_2_ values. Note that the inflection point in the $\dot{V}$ _E_-V_T_ relationship occurs at different V_T_ values, especially when comparing Arm+leg_TTE75_ and Leg_TTE75_ with the other four performance tests. A clear dissociation between *f*_R_ and $\dot{V}$ CO_2_ responses is observed for *f*_R_ values above 40 breaths min^−1^.

**FIGURE 8:**
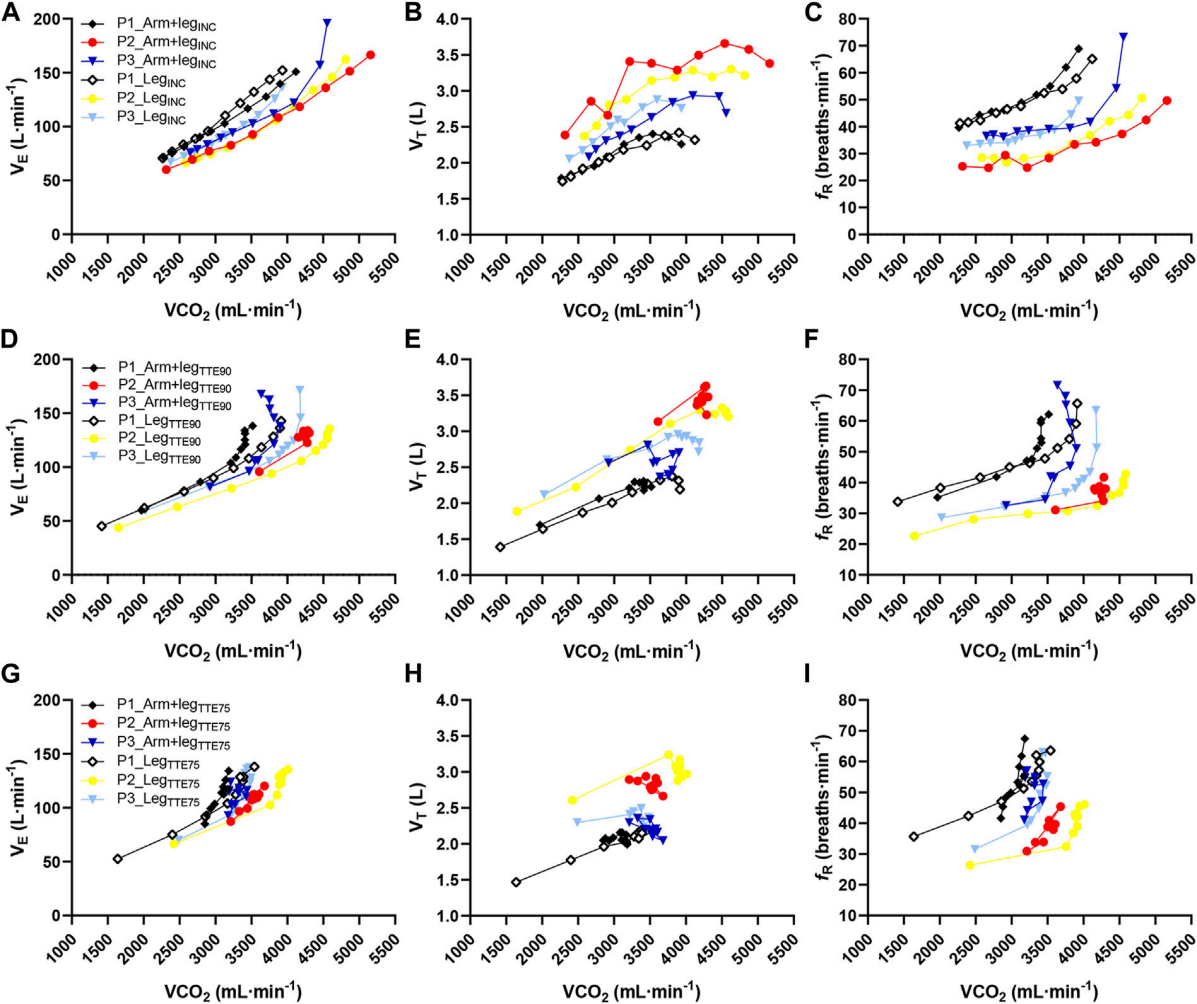
Relationship between minute ventilation and carbon dioxide output panels **(A,D,G)**, tidal volume and $\dot{V}$ CO_2_ **(B,E,H)**, and respiratory frequency and $\dot{V}$ CO_2_ **(C,F,I)** for three participants (named P1, P2, and P3) showing substantial interindividual differences in the breathing pattern. The Arm+leg tests are represented by black rhombi, red circles and dark blue reversed triangles for P1, P2, and P3 respectively, while the Leg tests are represented by open rhombi, yellow circles and light blue reversed triangles for P1, P2, and P3 respectively. Note that the shape of the relationship between minute ventilation and $\dot{V}$ CO_2_ shows some similarities with the relationship between *f*_R_ and $\dot{V}$ CO_2_ and that *f*_R_ and V_T_ show opposite responses when making interindividual comparisons.

Figure 8 shows the individual responses of $\dot{V}$ _E_, V_T_ and *f*_R_ expressed as a function of $\dot{V}$ CO_2_ values for three participants showing substantially different breathing patterns. The comparison between the responses of the three participants outlines how $\dot{V}$ _E_ is more closely associated with $\dot{V}$ CO_2_ than V_T_ and *f*_R_, and that higher values of *f*_R_ for a given $\dot{V}$ CO_2_ result in higher $\dot{V}$ _E_ values.

## 4 Discussion

This study aimed to systematically assess whether exercise tolerance improves with arm+leg cycling vs. leg cycling and whether *f*_R_ and perceived exertion are sensitive to the expected differences in exercise tolerance. This goal was achieved by comparing the two exercise modalities using three performance tests and two exercise paradigms (i.e. incremental test and TTE test). The main findings of the study are as follows: 1) exercise tolerance was substantially improved in all the Arm+leg tests; 2) perceived exertion, minute ventilation and respiratory frequency were particularly sensitive to the between-modality changes in exercise tolerance observed, unlike $\dot{V}$ O_2_ and heart rate. These findings support the notion that respiratory frequency is a marker of physical effort during high-intensity exercise and that its time course reflects changes in exercise tolerance. This holds true even during arm+leg cycling, which is an exercise modality where the responses of $\dot{V}$ O_2_ and heart rate do not reflect the reduction in physical effort and the improvement in exercise tolerance observed when comparing it with leg cycling.

Our findings provide convincing evidence that arm+leg cycling substantially increases exercise tolerance when compared to (conventional) leg cycling, hence expanding on the limited literature dealing with performance differences between the two exercise modalities (Secher et al., 1974; Bergh et al., 1976; Nagle et al., 1984). The apparent difference in the percentage improvement in exercise tolerance found between the incremental tests and the TTE tests is, in fact, in line with the different characteristics of the two performance paradigms. Indeed, a 1% improvement in power output in an incremental test, or in a time trial, results in a performance improvement that can exceed 10% in a TTE test (Hopkins et al., 1999). Hence, the average increase in power output of about 15% that we found in the incremental test is compatible with the average increase in TTE found in the TTE tests (i.e. 108% in the Arm+leg_TTE90_ and 90% in the Arm+leg_TTE75_). Such an improvement in exercise tolerance observed in the Arm+leg tests implies that the effort required to sustain a given power output is substantially lower at isotime compared to that of the Leg tests, and this premise is supported by our findings.

The notion that physical effort was lower in the Arm+leg tests than in the Leg tests is substantiated by the lower values of perceived exertion and *f*_R_ generally found at isotime. While the decrease in perceived exertion during incremental Arm+leg did not reach statistical significance (*p* = 0.057), it was substantial when comparing Arm+leg vs. Leg during the TTE tests. Indeed, perceived exertion was found to be among the most sensitive variables to variations in exercise tolerance during the TTE tests. These findings extend previous findings showing a lower RPE during arm+leg cycling vs. leg cycling at submaximal work rates (Hoffman et al., 1996; Hill et al., 2018). At isotime, *f*_R_ was significantly lower (*p* < 0.003; *η*_P_^*2*^ > 0.40) in the three Arm+leg tests than in the Leg tests, although this difference generally reached statistical significance in the last 20% of the Leg tests. This is an important feature of the *f*_R_ response that might have been missed in previous studies that did not compare Arm+leg and Leg until exhaustion (Robertson et al., 1986). Likewise, the reduction in *f*_R_ that is observed in the last part of a TTE test when exercise tolerance improves may not appear if the variability in TTE is not addressed on an individual basis when analyzing TTE data (Nicolò et al., 2019). We have overcome this problem by using the previously described “individual isotime” analysis (Nicolò et al., 2019), which reduces extensively the data loss that occurs when using more traditional analyses. Our findings collectively suggest that the time course of *f*_R_ reflects changes in exercise tolerance both during incremental exercise and TTE exercise, thus supporting the study hypothesis.

The association found between *f*_R_ and RPE and the sensitivity of *f*_R_ to changes in exercise tolerance support a substantial modulation of *f*_R_ by central command (Nicolò and Sacchetti, 2023). However, a partial dissociation was found between *f*_R_ and RPE, which may suggest that also muscle afferent feedback contributed to the *f*_R_ response. Although evidence suggests that muscle afferent feedback has a greater relative contribution to *f*_R_ during moderate exercise than during high-intensity exercise (Amann et al., 2010; Dempsey et al., 2014; Girardi et al., 2021; Nicolò and Sacchetti, 2023), arm+leg cycling implies the simultaneous use of the muscle mass of both the upper and lower limbs, possibly resulting in a greater magnitude of muscle afferent feedback, and especially of its mechanosensitive component (i.e. mechanoreflex). This may explain the slightly higher *f*_R_ shown in the first part of the TTE tests in the Arm+leg vs. the Leg modality. Indeed, it has been suggested that the relative contribution of muscle afferent feedback to ventilation is higher when the muscle mass recruited is larger (Amann et al., 2011; Dempsey et al., 2014; Nicolò and Sacchetti, 2023). Conversely, it is conceivable that the magnitude of the metabosensitive component of muscle afferent feedback (i.e. metaboreflex) was reduced at isotime in the Arm+leg tests because of the lower intramuscular metabolic perturbation. Hence, the metaboreflex cannot be ruled out as an input contributing to the decrease in *f*_R_ observed in the Arm+leg tests. Nevertheless, the relative contribution of muscle afferent feedback may reduce over time during a TTE test because the contribution of other inputs increases substantially (e.g. central command) (Dempsey et al., 2014; Nicolò and Sacchetti, 2023). We cannot exclude that afferent feedback from pulmonary mechanoreceptors or alterations in chest wall mechanics might have contributed to the partial dissociation observed between the *f*_R_ and RPE responses. Arm movements may increase the contribution to ventilation of afferent feedback from pulmonary mechanoreceptors or alter the mechanics of breathing, although these propositions require further investigation. Furthermore, it cannot be excluded that the lower pedaling cadence observed in the Arm+leg tests might have contributed to the between-modality differences observed in *f*_R_, although variations in pedaling cadence and *f*_R_ might not be proportional, especially during high-intensity exercise (Girardi et al., 2021). Conversely, it is less plausible that metabolic acidosis or other metabolic inputs might have provided a substantial direct contribution to the *f*_R_ modulation, and the reader is referred to previous studies where evidence supporting this proposition has been reviewed (Nicolò et al., 2020a; Nicolò and Sacchetti, 2023).

The ventilatory responses observed in this study can be interpreted in the light of a recently proposed model of ventilatory control during exercise, which suggests that *f*_R_ and V_T_ are modulated to a large extent by behavioral and metabolic inputs respectively (Nicolò and Sacchetti, 2023). While it was proposed that ventilation is differentially regulated during incremental exercise and TTE exercise (Syabbalo et al., 1994), our findings suggest that the breathing pattern is affected by the magnitude of the inputs modulating ventilation rather than by the type of exercise paradigm. Syabbalo et al. (1994) observed a more rapid and shallow breathing pattern during a TTE test at about 76% of the PPO reached in an incremental exercise than during this latter test. The lower V_T_ reported by Syabbalo et al. (1994) during the TTE test is in line with the V_T_ response observed during the TTE tests at 75% of the PPO_Leg_ in this study. However, the breathing pattern we observed in the TTE tests at 90% of the PPO_Leg_ was, conversely, more similar to that found during the incremental exercise than during the TTE tests at 75% of the PPO_Leg_ (see Figure 7). Hence, the rapid and shallow breathing pattern is not a feature of TTE exercise, which is in contrast with what Syabbalo et al. (1994) had suggested. Conversely, the observed findings can be explained by the differential control of *f*_R_ and V_T_ (Nicolò et al., 2017a; Nicolò et al., 2018; Nicolò and Sacchetti, 2019; Nicolò and Sacchetti, 2023). While *f*_R_ generally shows similar peak values during incremental exercise and constant work rate exercise (Syabbalo et al., 1994; Nicolò and Sacchetti, 2023), the V_T_ peak reached during exercise is largely influenced by the magnitude of metabolic inputs and is generally associated with the $\dot{V}$ CO_2_ peak (Nicolò and Sacchetti, 2023). As such, we found considerably lower V_T_ and $\dot{V}$ CO_2_ peak values in the Arm+leg_TTE75_ and Leg_TTE75_ tests than in the incremental tests. Conversely, when the difference in $\dot{V}$ CO_2_ peak between the incremental tests and the TTE tests was greatly reduced (i.e. when comparing the incremental tests with the Arm+leg_TTE90_ and Leg_TTE90_), the difference in V_T_ peak values decreased accordingly. Although an association between V_T_ peak and $\dot{V}$ CO_2_ peak is commonly found when considering different exercise conditions, populations and levels of exercise capacity (Nicolò and Sacchetti, 2023), the relationship between V_T_ and $\dot{V}$ CO_2_ is not always proportional because the V_T_ response is to some extent influenced by the *f*_R_ response (see discussion below). Furthermore, we acknowledge that V_T_ is influenced by various metabolic inputs that have not been measured in this study, including metabolic acidosis (Nicolò and Sacchetti, 2023).

The association between $\dot{V}$ CO_2_ and V_T_ helps explain why $\dot{V}$ _E_ resulted to be more sensitive than *f*_R_ to changes in exercise tolerance in this study. Indeed, both the magnitude of central command and that of metabolic inputs were probably higher in the Leg tests at isotime, thus increasing *f*_R_ and V_T_ respectively. However, the interpretation of the *f*_R_ and V_T_ responses observed requires careful consideration of the interdependence between the two components of $\dot{V}$ _E_, which has been advocated to explain the close match between alveolar ventilation and metabolic requirements (Haouzi, 2014). Substantial evidence suggests that V_T_ is fine-tuned based on *f*_R_ levels and the magnitude of metabolic inputs (Nicolò and Sacchetti, 2023), and this notion is reinforced by the present findings. While Figure 7 generally shows a consistent increase in V_T_ with increases in $\dot{V}$ CO_2_, the responses of the two variables diverge (i.e. V_T_ stabilizes or even decreases) when *f*_R_ starts to increase at a much steeper rate compared to $\dot{V}$ CO_2_. Notably, the V_T_ plateau did not occur at specific values of V_T_ or $\dot{V}$ CO_2_, and this is especially evident when considering the TTE tests at 75% of PPO_Leg_, where the steeper increase in *f*_R_ occurred at relatively low $\dot{V}$ CO_2_ levels. Although it has been proposed that the V_T_ plateau that occurs during high-intensity exercise depends on mechanical constraints (Jensen et al., 1980), evidence suggesting this proposition is scarce (Nicolò et al., 2020a; Nicolò and Sacchetti, 2023). Conversely, evidence suggesting that the stabilization of V_T_ depends to a large extent on the increase in *f*_R_ is substantial (Nicolò et al., 2020a; Nicolò and Sacchetti, 2023), and it is even more convincing during TTE tests performed at relatively low intensities, where pulmonary mechanical limitations in healthy individuals may not occur (Nicolò et al., 2020a; Nicolò and Sacchetti, 2023).

Individual responses further suggest that V_T_ may not change proportionally to $\dot{V}$ CO_2_ values because it is affected by *f*_R_ values. At given $\dot{V}$ CO_2_ levels, individuals with lower *f*_R_ values show higher V_T_ values and *vice versa* (see Figure 8). Different combinations of *f*_R_ and V_T_ may guarantee the match between alveolar ventilation and metabolic requirements, and the $\dot{V}$ _T_ fine-tuning feature is supposed to facilitate such a link (Nicolò and Sacchetti, 2023). Hence, individual responses reveal that $\dot{V}$ _E_ is more closely associated with $\dot{V}$ CO_2_ than V_T_, as also found in other exercise protocols (Nicolò et al., 2018; Girardi et al., 2021). The ability of the ventilatory control system to adjust V_T_ according to changes in *f*_R_ has nicely been shown both at rest and during exercise in studies replacing spontaneous breathing with different levels of voluntarily imposed *f*_R_ (Lamb et al., 1965; Kennard and Martin, 1984; Haouzi and Bell, 2009; Ohashi et al., 2013; Nicolò and Sacchetti, 2023). Conversely, the ventilatory control system appears not to match metabolic requirements effectively when V_T_ is imposed and *f*_R_ is free to vary (Ohashi et al., 2013). In this perspective, V_T_ may counteract interindividual differences in *f*_R_ and guarantee an appropriate match between alveolar ventilation and $\dot{V}$ CO_2_ for any values of *f*_R_ (Nicolò and Sacchetti, 2023). Our findings reveal the potential of comparing different exercise modalities and paradigms to shed light on the *f*_R_ and V_T_ modulation during high-intensity exercise.

The cardiocirculatory adjustments that occur when exercising simultaneously with the upper and lower limbs may provide further mechanistic support to the improvement in exercise tolerance observed in the Arm+leg modality. Arm+leg cycling may result in a greater peak cardiac output than Leg cycling (Secher et al., 1974; Reybrouck et al., 1975), and the higher $\dot{V}$ O_2_peak found in the Arm+leg_INC_ vs. Leg_INC_ is in line with this notion. Hence, it is conceivable that during Arm+leg_TTE90_ and Arm+leg_TTE75_ participants were exercising at a lower fraction of peak cardiac output compared to Leg_TTE90_ and Leg_TTE75_ tests respectively, especially when similar $\dot{V}$ O_2_ values were found across conditions at isotime. This may have contributed to accommodating the blood flow requests of both arm and leg muscles, thus improving muscle perfusion. Indeed, the reduction in the leg power output observed in the Arm+leg TTE tests vs. the Leg TTE tests of about 20% may have reduced the leg blood flow demand in the Arm+leg modality. In turn, the lower demand of the legs may have delayed the development of leg muscle fatigue and the increase in the magnitude of central command, thus contributing to the improvement in exercise tolerance observed in the Arm+leg modality. While it has been shown that the addition of (intense) arm work to leg work reduces the leg blood flow observed at a given leg power output (Secher et al., 1977; Secher and Volianitis, 2006), the relatively low intensity sustained by the arms in our study may have not impaired leg blood flow substantially.

The fact that *f*_R_ and $\dot{V}$ _E_ are considerably more sensitive to changes in exercise tolerance than $\dot{V}$ O_2_ and HR is particularly evident from the present study. Neither $\dot{V}$ O_2_ nor HR showed lower values in the Arm+leg tests than in the Leg tests at isotime, despite the lower physical effort and the improved exercise tolerance found in the Arm+leg modality. This is not surprising considering that $\dot{V}$ O_2_ is to a large extent associated with absolute power output during endurance cycling, although it also depends on other factors, including metabolic efficiency, which is lower for arm cycling compared to leg cycling (Cotes et al., 1969; Vokac et al., 1975; Louhevaara et al., 1990; Itoh et al., 2002). However, only a relatively small portion of the total power output is sustained by the arms during arm+leg cycling, and the oxygen uptake of arm+leg cycling has been reported to be minimally higher than that of leg cycling for a given power output (Hoffman et al., 1996). In our study, the addition of arm work to leg work resulted in a slightly higher or similar $\dot{V}$ O_2_ in the Arm+leg tests compared to the Leg tests. Likewise, similar or slightly higher values were observed for HR in the Arm+leg modality at isotime, and a higher maximal HR was observed in all the Arm+leg tests. In line with our findings, previous studies had raised concerns about HR monitoring during arm+leg cycling because of the different values of maximal HR and HR relative to RPE/% $\dot{V}$ O_2_peak that are observed when this modality is compared to leg cycling (Kitamura et al., 1981; Hoffman et al., 1996). Hence, the prescription and monitoring of arm+leg cycling should take this HR response into consideration and may benefit from the concomitant measurement of breathing variables (especially *f*_R_), which is technically feasible even in applied settings (Massaroni et al., 2019; Nicolò et al., 2020b).

The between-modality comparison of exercise tolerance, perceived exertion and $\dot{V}$ O_2_ shows important practical implications of exercising in the Arm+leg modality. Our findings suggest that this exercise modality allows individuals to nearly double the amount of time spent at a given $\dot{V}$ O_2_ during constant work rate exercise, or, by extension, to exercise at a higher $\dot{V}$ O_2_ for the same exercise duration and perceived exertion. This consideration is particularly relevant for exercising individuals interested in maximizing energy expenditure, for those willing to maximize the cardiometabolic stimulus of exercise, and for those interested in lowering effort for a given absolute cardiometabolic stimulus (Hill et al., 2018). Indeed, arm+leg cycling has the potential to increase exercise adherence because a high perceived exertion is commonly viewed as one of the main barriers to exercise participation (Cheval and Boisgontier, 2021), and a relatively low perceived exertion may be associated with a sufficient cardiometabolic stimulus in this exercise modality. Furthermore, considering that exercise tolerance is closely associated with morbidity and mortality (Kokkinos et al., 2010; Nesti et al., 2020), arm+leg cycling may have clinical implications. Arm+leg cycling involves the simultaneous use of arm and leg muscles and may result in a time-efficient training strategy for enhancing both health and performance (Zinner et al., 2017). However, further research is needed to test these propositions.

It is worth mentioning that the Arm+leg modality poses some measurement challenges that we had to face in this study. First, we did not attempt to compute the GET and the RCP because of the relatively high initial stage of the incremental tests imposed by the ergometer (i.e. 150 W), which has limited their detection. As such, the TTE tests were not prescribed based on exercise intensity domains. Hence, especially in the Arm+leg_TTE75_ test, it is possible that some participants were exercising in the severe intensity domain while others were in the heavy domain. Second, the measurement of blood lactate is very challenging during the Arm+leg modality, and we decided not to collect it to avoid interfering with the performance tests. Third, perceived exertion can only be rated verbally during the Arm+leg tests, thus generating breathing artifacts and affecting gas exchange measures. However, we used a filtering technique (Lamarra et al., 1987) that addressed this limitation and helped preserve the integrity of the physiological responses. Even the other limitations outlined were partially counteracted by the experimental design and the method of analysis used. Indeed, we performed a detailed between-modality comparison of the responses of some of the main physiological variables commonly used to compute the GET and the RCP. This comparison reveals a greater metabolic perturbation at isotime in the Leg tests, especially when considering the time courses of $\dot{V}$ _E_, $\dot{V}$ CO_2_ and P_ETCO2_. Hence, it is conceivable that participants were exercising at a lower relative exercise intensity during the Arm+leg tests, not only from an effort perspective but also from a metabolic perspective.

## 5 Conclusion

This study shows that exercise tolerance is substantially higher in the Arm+leg modality than in the leg cycling modality. The average improvement in exercise tolerance was 15% in the incremental test and 108% and 90% in the TTE tests at 90% and 75% of PPO_Leg_ respectively. Perceived exertion, minute ventilation and respiratory frequency were among the most sensitive variables to the improvement in exercise tolerance provided by the Arm+leg modality, hence suggesting that the common mechanism modulating these variables (i.e. central command) plays an important role in endurance performance. These findings reinforce the notion that respiratory frequency is a better marker of physical effort than oxygen uptake and heart rate. Our results have implications for devising exercise strategies to reduce perceived exertion while maintaining a high cardiometabolic demand, and for maximizing energy expenditure for the same level of effort exerted.

## Data Availability

The raw data supporting the conclusion of this article will be made available by the authors, without undue reservation.

## Abbreviations

Arm+leg, arm+leg cycling; Arm+leg_INC_, arm+leg cycling incremental test; Arm+leg_TTE75_, Arm+leg TTE test at 75% of PPO_Leg_; Arm+leg_TTE90_, Arm+leg TTE test at 90% of PPO_Leg_; *f*_R_, respiratory frequency; GET, gas exchange threshold; HR, heart rate; Leg, leg cycling; Leg_INC_, leg cycling incremental test; Leg_TTE75_, Leg TTE test at 75% of PPO_Leg_; Leg_TTE90_, Leg TTE test at 90% of PPO_Leg_; P_ETCO2_, end-tidal partial pressure of carbon dioxide; PPO, Peak power output; PPO_Leg_, peak power output reached in the Leg_INC_; RCP, respiratory compensation point; RPE, ratings of perceived exertion; TTE, time-to-exhaustion; $\dot{V}$ CO_2_, carbon dioxide output; $\dot{V}$ _E_, minute ventilation; $\dot{V}$ O_2_, oxygen uptake; $\dot{V}$ O_2_peak, peak value of oxygen uptake; V_T_, tidal volume.
